# Determination of Cyanide in Blood for Forensic Toxicology Purposes—A Novel Nci Gc-Ms/Ms Technique

**DOI:** 10.3390/molecules26185638

**Published:** 2021-09-17

**Authors:** Marcin Osak, Grzegorz Buszewicz, Jacek Baj, Grzegorz Teresiński

**Affiliations:** 1 Chair and Department of Forensic Medicine, Medical University of Lublin, Jaczewskiego 8b, 20-090 Lublin, Poland; marcin.osak@umlub.pl (M.O.); grzegorz.buszewicz@umlub.pl (G.B.); grzegorz.teresinski@umlub.pl (G.T.); 2 Chair and Department of Human Anatomy, Medical University of Lublin, Jaczewskiego 4, 20-090 Lublin, Poland

**Keywords:** cyanide, blood, cyanide in blood, gas chromatography-mass spectrometry, GC-MS, GC-MS/MS, toxicology, analytical method, forensic science, poisoning investigation

## Abstract

One of the recently evolving methods for cyanide determination in body fluids is GC-MS, following extractive alkylation with pentafluorobenzyl bromide or pentafluorobenzyl *p*-toluenesulfonate. The aim of this study was to improve previous GC methods by utilizing a triple quadrupole mass spectrometer, which could enhance selectivity and sensitivity allowing for the reliable confirmation of cyanide exposure in toxicological studies. Another purpose of this study was to facilitate a case investigation including a determination of cyanide in blood and to use the obtained data to confirm the ingestion of a substance, found together with a human corpse at the forensic scene. The blood samples were prepared following extractive alkylation with a phase transfer catalyst tetrabutylammonium sulfate and the PFB-Br derivatization agent. Optimal parameters for detection, including ionization type and multiple reaction monitoring (MRM) transitions had been investigated and then selected. The validation parameters for the above method were as follows—linear regression R^2^ = 0.9997 in the range of 0.1 µg/mL to 10 µg/mL; LOD = 24 ng/mL; LOQ = 80 ng/mL and an average recovery of extraction of 98%. Our study demonstrates the first attempt of cyanide determination in blood with gas chromatography-tandem mass spectrometry. The established method could be applied in forensic studies due to MS/MS confirmation of organic cyanide derivative and low matrix interferences owning to utilizing negative chemical ionization.

## 1. Introduction

Cyanogenic compounds in the toxic forms of hydrogen cyanide and its salts constitute a significant research task in the field of forensic toxicology. Being a highly bioavailable chemical, cyanide is easily absorbed through the skin and mucosa into the body fluids where it is further distributed to the tissues exerting its acute effects on the vital organ systems [1,2,3]. When cyanide is introduced into the cells, it binds to and inactivates multiple enzymes containing transition metals, essential for cellular metabolism, and proper regulation of ionic homeostasis. Among the many interactions produced by cyanide, the inhibition of cytochrome C oxidase is the primary mechanism underlying histotoxic hypoxia and acute toxicity [3,4]. Since the organs with the greatest oxygen demand such as the brain and heart, are most sensitive to cyanide acting, manifestations of the poisoning are associated with the disorders of basic vital functions and occur rapidly after exposure [5,6].

The most common sources of cyanide exposure include inhalation of smoke from the nitrogen-containing materials [7,8,9], accidental exposure to dust or fumes during industrial operations, nitroprusside therapy, delivery of plant glycosides with food (e.g., cassava), intentional homicides, and voluntarily ingestion in suicides, usually in the form of a salt (KCN, NaCN) [3,10]. Despite distinct toxicity attributed to the cyanide, its low doses are effectively detoxified to thiocyanate, primarily via a rhodanese-mediated reaction with sulfur donors as substrates. Thiocyanate is much less toxic than cyanide and is excreted with urine [5,10]. When acute poisoning occurs, cyanide concentration reaches the saturation state of the detoxification pathways, resulting in a slowed metabolism to less toxic compounds such as thiocyanate. Irreversible hypoxia contributes to the steep dose-response and impairing of neurological, cardiovascular, and respiratory functions [3,6,10].

Methods that are currently mostly applied for the determination of cyanide in biological fluids include a variety of techniques as spectrophotometric assays, gas chromatography-flame ionization detection (GC-FID), gas chromatography-electron capture detection (GC-ECD), gas chromatography-nitrogen-phosphorus detection (GC-NPD), gas chromatography mass spectrometry (GC-MS), and liquid chromatography-tandem mass spectrometry (LC-MS/MS) [11,12,13,14]. Detection of cyanide could be also performed by ion-mobility spectrometry (IMS), a technique frequently combined with headspace-gas chromatography (HS-GC) to determine volatile and semi-volatile compounds. IMS is also used alone as a real-time simple technique, able to identify and quantify trace amounts of vapors in the atmosphere [15]. For the gas chromatography, cyanide determination could be performed via headspace injection of vapors containing hydrogen cyanide or liquid injection of extract containing cyanide derivative. However, direct HCN determination by the headspace gas chromatography-mass spectrometry (HS-GC-MS) has limited detector performance caused by a low molar mass of target [16]. Another limitation related to the usage of a headspace technique is poor retention of hydrogen cyanide on the gas chromatography (GC) columns and the necessity of usage of a cryogenic trap or a split injection in order to obtain a properly resolved peak [17,18]. Those drawbacks could be prevented when cyanide is derivatized to a higher mass molecule, allowing for a sensitive detection with mass spectrometry and easier separation on the GC capillary column [16]. From among proposed techniques with derivatization of cyanide ion, GC appears more efficient than HPLC in terms of higher column resolution, simpler sample preparation and high detection selectivity owning to properties of target compound PFB-CN, favorable for identification with mass spectrometry [16,19]. Moreover, the reaction of pentafluorobenzylation, by which a highly reactive cyanide ion is incorporated into a structure of organic nitrile, improves cyanide stability for common storage conditions [20]. The demonstrated method utilizes sample preparation conditions such as alkaline stock solutions and alkaline tetrabutylammonium sulfate (TBAS) buffer which prevent cyanide loss from a sample by the generation of volatile hydrogen cyanide. The solutions of 50 mM sodium hydroxide (pH 12.7) and phase transfer catalyst buffer TBAS (pH 9.45) utilized in the method have pH above HCN pKa value (pH 9.21) [10].

The determination of cyanide pentafluorobenzyl derivatives in biological samples is mainly performed with either GC-ECD or GC-MS systems [21,22]. However, in the currently reported GC-MS methods that enable cyanide detection after pentafluorobenzylation, single quadrupole detectors were utilized which have considerable selectivity limitations in comparison to triple quadrupole mass spectrometers. Moreover, previous GC-MS methods of analyzing cyanide-PFB in blood utilized mainly electron ionization (EI) or positive chemical ionization (PCI), which commonly are less efficient in halogens detection than negative chemical ionization (NCI) [23]. Chemical modification of cyanide ion into pentafluorobenzyl cyanide results in the formation of a more complex organic compound. This modification narrows down the range of tools that allow for accurate confirmation of the structure of pentafluorobenzyl cyanide (PFB-CN) as a molecular target. The above requirement prompted us to validate a new technique basing on tandem mass spectrometry, which poses a desirable tool in the forensic toxicology context.

The purpose of this study was to develop a new analytical method of cyanide determination in biological samples. In this study, we examined the conditions and parameters of the reported GC-MS methods and attempted to apply a triple quadrupole mass spectrometer and negative chemical ionization.

## 2. Methods

### 2.1. Materials

**Figure 1:**
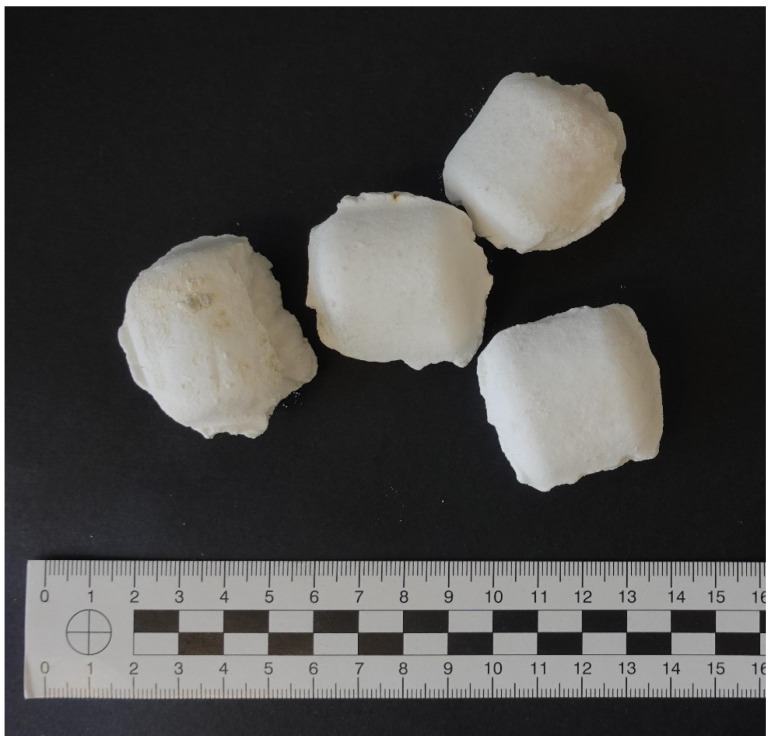
Substance discovered near cadaver, subjected to evaluation for cyanide in its composition.

The biological material used to develop our method was the blood from a group of healthy donors and blood spiked with the known amount of potassium cyanide standard. The test sample was the blood obtained from a deceased individual suspected of cyanide poisoning, delivered to the Chair and Department of the Forensic Medicine in Lublin in order to determine the cause of death. The forensic evidence also included a non-identified substance in the form of white briquettes, discovered together with a cadaver (Figure 1).

A medical examination of the deceased revealed the changes within the body surface in the form of cherry red livor mortis, suggesting a possible cyanide poisoning. Blood aimed to be tested and reference blood samples collected from non-exposed individuals were stored in the freezer at −20 °C to maintain their composition until the analysis.

This study was performed in accordance with institutional and national regulations and approved by the Bioethics Committee of Medical University of Lublin (ethics approval number: KE-0254/32/2019).

### 2.2. Chemicals

Potassium cyanide ACS reagent (≥96.0%) was purchased from Sigma-Aldrich^®^ (St. Louis, MO, USA); isotopically labeled potassium cyanide K^13^C^15^N (99% 13C, 98% 15N) from Aldrich^®^ (Milwaukee, WI, USA); the phase transfer catalyst, tetrabutylammonium sulfate solution 50 wt.% in H_2_O from Aldrich^®^; di-sodium tetraborate decahydrate from Merck KGaA^®^ (Darmstadt, Germany); an alkylating agent, pentafluorobenzyl bromide (PFB-Br), from Supelco^®^ (Bellefonte, PA, USA); sodium hydroxide solution 50% from Merck KGaA^®^; sodium sulfate from Merck KGaA^®^; ethyl acetate CHROMASOLV^TM^ LC-MS from Honeywell^®^ (Morris Plains, NJ, USA); water CHROMASOLV^TM^ LC-MS from Honeywell^®^ and an ethylbenzenesulfonic silica gel column (HyperSep SCX, 100 mg) from Thermo Fisher Scientific^®^ (Waltham, MA, USA).

### 2.3. Biohazard Information

Cyanide salts e.g., potassium and sodium cyanide are highly toxic and reactive substances. It is strongly recommended to use a well-ventilated hood while operating with those substances and avoid contact with the acidic solutions in order to prevent the generation of volatile and extremely toxic hydrogen cyanide. Besides, pentafluorobenzyl bromide should be handled with particular caution since it strongly irritates the eyes, skin, and mucosa, leading to breathing problems, skin burns, and even eye damage.

### 2.4. Sample Preparation

Stock standard solutions were prepared by dissolving potassium cyanide in 50 mM NaOH, which stabilizes the ionic form of cyanide. Calibration solutions were made by further dilutions with the same medium. Calibration samples were prepared by immediate adding 25 µL of IS (^13^C^15^N^−^) and 25 µL of proper standard solution (CN^−^) to 100 µL of the reference blood. Samples to be tested were prepared by adding 25 µL of IS (^13^C^15^N^−^) at concentration of 4 µg/mL and 25 µL of 50 mM NaOH to 100 µL of the examined blood. Utilization of NaOH solutions in our method provides stability of ionic cyanide form in blood specimens. The mixture of the blood and a standard was vortex-mixed for 10 s. Then, 600 µL of 10 mM TBAS (dissolved in a saturated solution of the disodium tetraborate decahydrate) was added, along with 600 µL of 20 mM PFB-Br (derivatization agent) in anhydrous ethyl acetate. The extraction was performed via vortex-mixing for 45 s and derivatization by incubating samples at 65 °C for 30 min. After a derivatization, the samples were centrifuged (15,000 rpm/10 min/10 °C). The upper layer of the organic phase (600 µL) was separated and passed through the solid phase extraction (SPE) HyperSep SCX columns. Finally, the extracted samples were collected to Eppendorf vials with 40 mg anhydrous sodium sulfate, vortex-mixed, and centrifuged (14,000 rpm/2 min). The prepared extract was placed in chromatographic vials and subsequently analyzed with the gas chromatography-tandem mass spectrometry (GC-MS/MS) system.

### 2.5. Instrumentation

Cyanide in the form of alkylated derivative PFB-CN was analyzed using a Trace 1310 Gas Chromatograph combined with TSQ 8000 EVO Triple Quadrupole Mass Spectrometer (Thermo Fisher Scientific^®^, Waltham, MA, USA). The samples were injected at a volume of 1 μL in the splitless mode with a 0.75 min valve-off time and a constant temperature of 210 °C. The GC oven was equipped with Rxi-5ms fused silica capillary column (30 m × 0.25 mm internal diameter, 0.25 µm film thickness) coated with 5% phenyl methyl silicone as a stationary phase. The carrier gas used was helium, with a constant flow of 1 mL/min. The temperature gradient of chromatography was programmed as follows: 40 °C (0.8 min hold) increased to 130 °C at 15 °C/min (0.2 min hold) and further increased to a final value of 250 °C at 30 °C/min (1.5 min hold). Starting chromatography at 40 °C was essential to obtain the suitable peak symmetry on the grounds that the applied GC column retains PFB-CN via solvent effect (when ethyl acetate is used as a medium). Alkyl derivatives were identified by GC-MS/MS in positive (CI^+^), negative (CI^−^) chemical ionization, and electron ionization (EI). The optimization of detection was performed by examining of the above ionization types as candidates. MS/MS transitions for each ionization technique were studied with the Auto SRM tool TraceFinder GC (Thermo Fisher Scientific^®^, Waltham, MA, USA). Fragmentation products were investigated in a full range of collision energies. In the validated method, the detection was performed by multiple reaction monitoring mode. Methane was used as an ionization gas at a flow rate of 1 mL/min. The temperatures of the transfer line and the ion source were 260 °C and 250 °C, respectively. Argon was utilized as the collision gas.

### 2.6. Validation

GC-MS/MS method validation was performed in accordance with the FDA guidelines for the bioanalytical method [24]. The determined parameters of the method included selectivity, linearity, sensitivity, accuracy, precision, recovery, matrix effect, and stability. The selectivity of the method was evaluated by analyzing ten (n = 10) reference samples and quantitation the endogenous level of cyanide. The method was tested for interferences of the matrix at the retention times (Rt) corresponding to an analyte and internal standard. While specificity was assessed by the ability of the method to differentiate reference and elevated cyanide levels.

Linearity was expressed as linear regression and determined by preparing a calibration curve including five points at concentrations presented in Table 1.

The sensitivity of the method was assessed by calculation the limit of detection (LOD) and lower limit of quantitation (LLOQ). Reference blood samples also presented a slight chromatographic peak of PFB-CN in our method. Therefore, detection and quantitation limits were established basing on the calibration curve and values of cyanide concentration obtained in ten (n = 10) reference blood samples. LOD and LLOQ were calculated on the calibration curve slope and the standard deviation of the response, using following equations: LOD = 3σ/S and LLOQ = 10 σ/S, where σ = the standard deviation of the measured endogenous cyanide concentrations (n = 10), S = calibration curve slope.

The analyte recovery was calculated by comparing the area ratio (analyte to internal standard) of four QC samples (n = 3 at each concentration) at LLOQ, low (LQC), mid (MQC), and high (HQC) concentration of cyanide (0.1, 0.3, 1, 10 μg/mL) with recovery samples, prepared from blood extracts spiked with QC standard and incubated after the step of separating the organic phase and subsequently subjected for purification by SPE in normal order as described in the sample preparation procedure. The matrix effect was calculated by comparing the area ratio (PFB-CN to IS) between blood samples and water samples at the same four QC concentrations prepared in triplicate (0.1, 0.3, 1, 10 μg/mL).

The determination of accuracy and precision was performed by the analysis of different QC standards (0.1, 0.3, 1, 10 μg/mL) in a quadruplicate on three consecutive days. The stability of cyanide in the spiked blood at low and high concentrations was examined at −20 °C, 4 °C, and room temperature, and for three freeze-thaw sessions within the 7 days. The quantitation peak area ratio of the analyte to internal standard was used for calculation and comparison with freshly prepared samples.

## 3. Conclusions

### 3.1. Extraction Procedure

The previously developed GC-MS methods by Bhandari et al. [25] or Kudo et al. [20] also utilized the process of catalyzed pentafluorobenzylation to cyanide ion determination in the biological fluids, which turns out as an advantageous solution for reactive and low weight target. The aformentioned papers served as a fundament in developing a new improved technique and its successful application to toxicological assay that was demonstrated in our study. The helpful information was reported by Kudo et al. in a method that included the SPE step in sample preparation. This element was, in turn, omitted in a study performed by Bhandari et al., despite utilizing a similar extraction procedure with tertiary ammonium salt as a catalyst. We also attempted to analyze cyanide without the SPE step and as a result, serious chromatographic aberrations occurred. After only a few injections, we observed a broadening peak shape, shortening of retention time, and analyte carryover. It can, therefore, be assumed that tertiary ammonium salts e.g., TBAS remain in an organic phase after the derivatization and lead to the serious contamination of the GC inlet and column. By including SPE to our method, final extracts were free of TBAS catalyst and safe for analytical instrument efficiency and therefore provided method reproducibility during multiple sample analyses.

### 3.2. Ionization Technique Comparison

The derivatized compound in the form of pentafluorobenzyl cyanide (2,3,4,5,6-pentafluorophenylacetonitrile) yielded typical spectra for each ionization technique. Unlike the others, EI spectra showed next to molecular ion (207 Da) two fragmented ions containing -CN moiety (188 Da and 157 Da) along with other numerous ions with low signal intensity. In contrast, chemical ionizations revealed a lower tendency to ion fragmentation and clearer spectra of the target.

**Figure 2:**
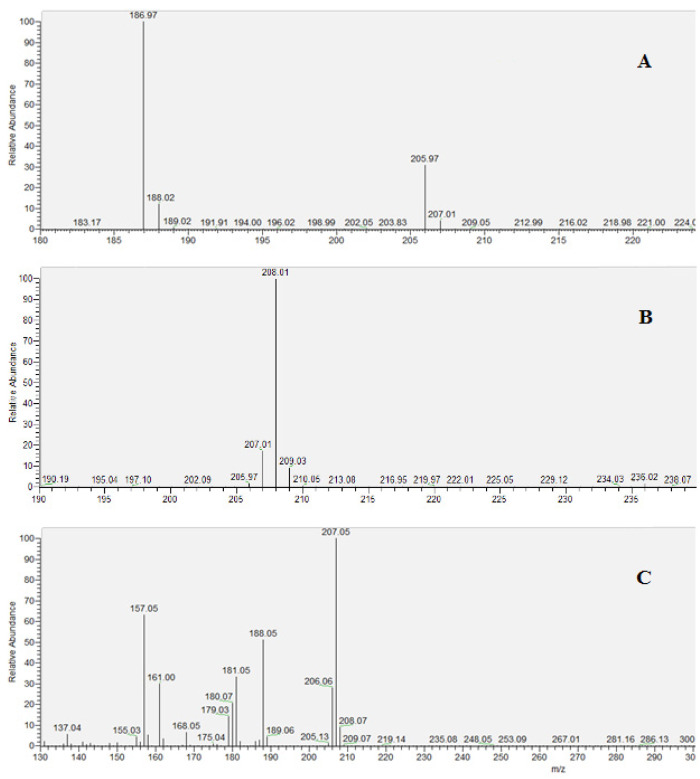
Mass spectra of the cyanide derivative PFB-CN in cyanide spiked blood samples, obtained with the following ionization techniques: (**A**) NCI, (**B**) PCI, (**C**) EI positive.

In the case of CI^+^ protonated molecular ion the peak at 208 Da was identified as cyanide-PFB and 210 Da as labelled cyanide-PFB. Whereas CI^−^ spectra showed mainly dissociated molecular ion for isotope-labelled cyanide-PFB with natural isotopic composition and isotopically enriched internal standard, as follows: PFB-CN–HF (187 Da), PFB-^13^C^15^N–HF (189 Da) (Figure 2).

### 3.3. MRM Optimization

In the case of PCI, the PFB-CN + H+ (208 Da) ion and PFB-13C15N + H+ (210 Da) ion were fragmented. The results obtained from the MRM study for positive chemical ionization, showed identical fragments for derivatives of cyanide and its ^13^C^15^N isotope, which means that the detected ions didn’t contain a -CN moiety. According to this observation, a suitable selectivity has not been achieved with the PCI technique for the monitored transitions. Moreover, the above ionization type had a relatively low signal intensity when compared with other ionizations. The next EI technique was tested by fragmenting ions 207 Da (molecular ion), 188 Da, and 157 Da of cyanide standard derivative, along with ions 209 Da (molecular ion), 190 Da, and 159 Da of the internal standard derivative. Despite producing ions, whose mass differences corresponded to those occurring between cyanide and its 13C15N isotope, we observed an unwanted susceptibility of this ionization type for biological matrix interferences. The quantitation performed on the blood samples was substantially limited by unacceptable linearity at lower calibration points, which is a serious disadvantage of the EI technique in a toxicological assay.

The most sensitive cyanide detection was achieved with negative chemical ionization (NCI) because it provided the highest signal-to-noise ratio when compared to a positive chemical as well as an electron ionization. Regarding the significant reduction of matrix interferences by a negative polarization, the analyte signal was clearly separated from the background, allowing for precise quantitation of the target compounds.

**Figure 3:**
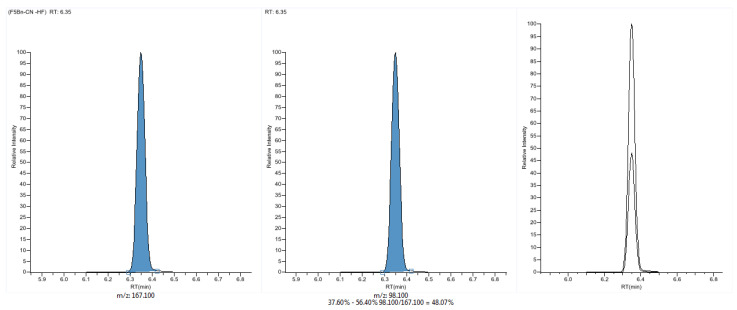
Mass chromatograms of PFB-CN obtained from cyanide spiked blood samples: quan peak 187→167.1, confirmation peak 187→98.1, and ion overlay chromatogram.

The NCI one turned out to be the most useful technique, because it produced unique ions (containing -CN moiety) for each compound in the target analyses and was the most resistant for matrix effects. The MRM detection includes two transitions of the analyte and internal standard presented in Table 2 and Figure 3.

### 3.4. Method Validation

The method demonstrated high selectivity due to the lack of interfering peaks at the retention times of the analyte and internal standard and showed repeatable retention times in the range of 6.35–6.38 min (±0.33%) over ten samples. The peak area ratio of quality ion to quantitation ion was in the range of 47–48.5% at the concentrations of cyanide covered by a calibration curve. Each blood sample presented a slight PFB-CN peak, probably of natural origin and all ten samples were quantitated. The average concentration of cyanide in reference samples was 43 ng/mL (with a standard deviation of 8.4 ng/mL) and all values were not higher than 60 ng/mL. The variation coefficient for the analyzed reference samples was not higher than permissible 20%. Obtained results showed that the method allows to differentiate nominal and elevated cyanide levels since the potentially toxic range is concentration 500 ng/mL–1 μg/mL [11,26].

The demonstrated method has acceptable sensitivity due to its low LOD and LLOQ values. The calculated limit of detection for the described method was set at 24 ng/mL and the limit of quantitation was set at 80 ng/mL. The calibration curve included five points and was linear in the range from 100 ng/mL to 10 μg/mL, which covers cyanide concentrations generally considered as harmful or lethal. The presented method presents excellent linearity with a correlation coefficient of 0.9997. The linear regression equation of PFB-CN calibration was y = 1.05x + 0.099. The results of validation studies regarding accuracy, precision, recovery, and matrix effect are summarized in Table 3.

All RSD values and coefficients of variation for accuracy and precision are well within the permissible ±15% error. The precision calculated as a percent relative standard deviation was below 5% in the intra-assay analysis and below 8% in the inter-assay analysis. The accuracy for intra-assay and inter-assay analyses was also acceptable with RSD not higher than 8% of nominal concentration. The average recovery of cyanide ion from the whole blood was 98%, which poses a satisfactory result for the toxicological assay.

Cyanide was stable after the storage at low temperatures when compared to the room temperature. After 7 days of storage, the percentage of the original cyanide concentrations in the samples for each condition were as follows, 96% for −20 °C and 83% for 4 °C. At room temperature, cyanide concentration decreased to 61% of a nominal concentration and after three sessions of the freeze-thaw experiment, the concentration decreased to 79% of the original value. Our experiments showed that cyanide stability was highly dependent on the storage conditions, wherein the biggest concentration change was observed at room temperature.

### 3.5. Method Application

The devised method was used in a toxicological analysis of the factual poisoning case. The blood samples were analyzed in quadruplicate and an average concentration was 21.54 μg/mL (%RSD = 2.16), which is above the methods ULOQ (10 μg/mL). The determined cyanide in the blood proves a lethality of a suspected poisoning case as the concentrations above 2 μg/mL are generally considered as potentially life-threatening [26]. Besides, the white briquettes collected at the scene of the incident were evaluated qualitatively by preparing solutions at a concentration of 10 μg/mL and applying the described technique, they were confirmed as a cyanide salt. The signal level of the PFB-CN peak in the analyzed material was well above triplicate of the background noise, in the range of 343–375.

## 4. Discussion

Cyanide poisoning can be confirmed with variety of techniques, different in terms of costs of implementation, time consumed, selectivity, evidential value, and method capacity. Despite availability of wide range of analytical tools cyanide poses a demanding target, because of its low half-life in samples stored at ambient temperature and susceptibility to interconversion of cyanide and its main metabolite thiocyanate, artifactual formation of cyanide and its disappearance caused by formation of volatile HCN [27,28]. Our results confirm previously reported observations from sample storage experiments. Cyanide concentration in blood sample was highly dependent on storage temperature and it highly decreased within seven days of storage in room temperature. In other studies, carried out on a few different biological materials, was showed up that changes in cyanide concentration are bidirectional which adds difficulties in predicting cyanide behavior in cadavers’ blood and tissues after a few days from incident. However, observed artifactual production of cyanide in samples from non-exposed groups was negligible within 24 h of death and didn’t reached levels corresponding to toxic or lethal thresholds. The recommendations based on the current studies are that all biological samples should be collected as soon as possible and shortly subjected for analysis. Alternatively, samples should be preserved by immediate storing in deep freezer at (−20 °C) to obtain reliable results. Samples should be analyzed or preserved within 24 h after death. Furthermore, the time intervals in each step between death and analysis should be noted to facilitate the interpretation of the results [27,28,29]. Another challenges in cyanide determination are related with method selectivity required in forensic toxicology field, which should allow for distinguish endogenous and elevated cyanide levels without impact of interferences. Therefore, we decided to validate method based on efficient analytical instrument GC-MS/MS. To increase sensitivity of detection for light cyanide molecule we applied derivatization step, which converted it to compound being within the detector’s optimal working range. On the other hand, derivatization of cyanide leads to the formation of a more complex compound, which imposes additional requirements on the analytical method in terms of identification accuracy. Therefore, as customary in forensic toxicology, it is reasonable to use a tandem mass spectrometry that allows for unique identification of compounds to provide outstanding sensitivity. By studying and evaluating parameters reported in the previous methods [11,13] we established a detection technique that seems to be more favorable and directed to halogens e.g., cyanide derivative (PFB-CN). Since the strongly electronegative fluoride atoms are constituent of our molecular target it tends to give a higher signal level in the negative polarization and offers the possibility of significant reduction of the unwanted noise. Our study confirmed the advantage of NCI over other techniques in biological matrices analyses. Applying MS/MS scanning type provided additional improvement of detection selectivity when compared to previously reported GC-MS settings. Comparing the new GC-MS method with the previous ones, there is a noticeable difference in the achieved limits of detection and quantitation. Bhandari et al. [23] reported a limit of detection 26 ng/mL and a limit of quantitation 260 ng/mL in plasma for PCI GC-MS, while Kudo et al. [18] reported a limit of detection 130 ng/mL and a limit of quantitation 260 ng/mL in the whole blood for the EI GC-MS method. Despite a more rigorous approach to establish these parameters for our method, these parameters were established as follows, the limit of detection 24 ng/mL and limit of quantitation 80 ng/mL. The test performed on a poisoning victim along with satisfactory validation parameters proves the success of the new NCI GC-MS/MS method in confirmation and quantitation of the exposure to cyanide in forensic cases. With improvements applied, the new method can pose an alternative tool in applications that emphasized reliability and sensitivity, particularly in the toxicological studies in forensic medicine institutes. Proposed method disadvantages are related mainly with high costs and time-consuming sample extraction, which limits the applicability in clinical settings appreciating fast techniques. However, the described technique with pentafluorobenzyl alkylation creates additional opportunities for unambiguous confirmation of other numerous anions, essential for toxicological or medical studies.

## Figures and Tables

**Table 1:** PFB-CN calibration levels.

Calibration Level	Cyanide Concentration (µg/mL)
1	0.1
2	0.4
3	1
4	4
5	10

**Table 2:** Parameters of MS/MS detection for pentafluorobenzyl cyanide natural isotope and isotopically-labelled internal standard.

Compound	Retention Time (min)	Precursor Ion (*m*/*z*)	Product Ion (*m*/*z*)	Collision Energy
F5Bn-CN (-HF)	6.35	187	98.1	25 eV
			167.1 (quantification ion)	10 eV
F5Bn-^13^C^15^N (-HF)	6.35	189	100.1	25 eV
			169.1	10 eV

**Table 3:** Extraction recovery, matrix effect, accuracy, and precision of cyanide-PFB in human blood at four QC concentrations (0.1, 0.3, 1, 10 μg/mL).

QC Concentration		Recovery	Matrix Effect	Intraday		Interday	
				Accuracy	Precision (% RSD)	Accuracy	Precision (% RSD)
LLOQ	100 ng/mL	110.4%	115.5%	97.7%	4.5	92.2%	6.8
LQC	300 ng/mL	98.3%	103%	95.1%	4.0	92.5%	6.6
MQC	1 μg/mL	96.5%	101.0%	101.4%	2.6	96.1%	3.9
HQC	10 μg/mL	88.2%	87.4%	100.7%	1.7	97%	2.7

## Data Availability

All data are within this manuscript.

## Sample Availability

Samples of the compounds are not available.
